# Traffic-Aware Secured Cooperative Framework for IoT-Based Smart Monitoring in Precision Agriculture

**DOI:** 10.3390/s22176676

**Published:** 2022-09-03

**Authors:** Ibrahim Abunadi, Amjad Rehman, Khalid Haseeb, Lorena Parra, Jaime Lloret

**Affiliations:** 1 College of Computer & Information Sciences, Prince Sultan University, Riyadh 11586, Saudi Arabia; 2 Artificial Intelligence and Data Analytics (AIDA) Lab, CCIS Prince Sultan University, Riyadh 11586, Saudi Arabia; 3 Department of Computer Science, Islamia College Peshawar, Peshawar 25000, Pakistan; 4 Instituto de Investigación para la Gestión Integrada de Zonas Costeras, Universitat Politenica de Valencia, 46022 Valencia, Spain

**Keywords:** agriculture system, Internet of Things, fog system, soil monitoring, green energy

## Abstract

In recent decades, networked smart devices and cutting-edge technology have been exploited in many applications for the improvement of agriculture. The deployment of smart sensors and intelligent farming techniques supports real-time information gathering for the agriculture sector and decreases the burden on farmers. Many solutions have been presented to automate the agriculture system using IoT networks; however, the identification of redundant data traffic is one of the most significant research problems. Additionally, farmers do not obtain the information they need in time, such as data on water pressure and soil conditions. Thus, these solutions consequently reduce the production rates and increase costs for farmers. Moreover, controlling all agricultural operations in a controlled manner should also be considered in developing intelligent solutions. Therefore, this study proposes a framework for a system that combines fog computing with smart farming and effectively controls network traffic. Firstly, the proposed framework efficiently monitors redundant information and avoids the inefficient use of communication bandwidth. It also controls the number of re-transmissions in the case of malicious actions and efficiently utilizes the network’s resources. Second, a trustworthy chain is built between agricultural sensors by utilizing the fog nodes to address security issues and increase reliability by preventing malicious communication. Through extensive simulation-based experiments, the proposed framework revealed an improved performance for energy efficiency, security, and network connectivity in comparison to other related works.

## 1. Introduction

Today, the Internet and the Internet of Things (IoT) dominate nearly everyone’s life. IoT is a paradigm that connects things, people, or networks and allows them to process and react precisely to any kind of physical or virtual communication [1,2,3]. IoT has applications in every industry, including healthcare, agriculture, and home controllers. It efficiently provides user-required services by utilizing Internet connectivity, sensors, and a variety of other technologies and protocols for collecting and analyzing data [4,5,6]. The Internet of Things helps companies to automate operations and improve service delivery using Internet technologies and cloud-based data transmission. For the various industries it is utilized in, IoT does not permit the adoption of universal software architecture; instead, it must be modified to meet user requirements [7,8,9]. Nowadays, smart agriculture is more important than ever because of the expanding global population and rising food demand. In this context, smart technologies have become a crucial route to cutting-edge agricultural practices [10,11,12]. There are many different applications, protocols, and prototypes in the field of agricultural land.

Furthermore, several IoT policies and standards have been developed in the agriculture sector in numerous nations and organizations worldwide. Due to the limited boundaries of IoT devices, sensors, and actuators, a detailed study of IoT in the context of agriculture is required to understand the present state of research [13,14,15]. Throughout history, the agricultural industry has played a significant role in human cultures worldwide. Machine learning is a subset of artificial intelligence widely explored for identifying malicious attacks. Such a technique also focuses on making the system smart so that additional overhead can be reduced on the IoT devices [16,17]. The organization and practices of modern agriculture are significantly impacted by the development of information and communication technologies (ICTs). Despite the benefits of this evolution, several security concerns have the potential to negatively affect the agriculture sector [18,19,20]. Since farmers could sustain a significant financial and personal loss in the event of a data breach, data integrity and confidentiality are critical security concerns within the agricultural sector [21,22,23]. In this work, the main contributions are as follows:

- It provides a strategy to effectively manage IoT resources, identify the redundant nodes that are collecting the same data and forbid using them. Such a scheme is efficient in utilizing the resources of the network and prolonging the system life cycle.
- It develops intelligent methods using distributed machine learning to predict routing decisions for the selection of optimal forwarders with increasing data delivery and load balancing.
- Another aspect of the proposed framework is the usage of a fog system to establish a secure chain in the presence of unidentified and faulty nodes by establishing a reliable group of nodes.
- We verified the proposed framework with other work and the results demonstrate enhanced performance for various network parameters.

This paper is further organized as follows: the discussion for related studies is presented in Section 2. Section 3 presents the detail of the proposed framework. In Section 4, the simulation environment is briefly explained. Section 5 presents the results and discussion of the experiments. Lastly, the conclusion is in Section 6.

## 2. Related Work

The smart city emerged as a concept with the fast growth of dependable information and communication models and the combination of sensor technologies [24,25,26]. Modern fog computing technology develops intelligent networks combining IoT networks and cloud platforms. Fog computing is applied at the network edge, and they perform a significant number of tasks in terms of processing, storage, and communication. Networking devices such as routers, gateways, etc., make up fog computing. Compared to sensor nodes, these devices have more processing, transmission, and storage capabilities [27,28]. In an IoT system, fog nodes received data from sensors and are further forwarded for high-cost processing with the support of data centers. A smart city promotes sustainability by utilizing various sensors to collect information from the environment while providing improved social facilities, transportation, and accessibility. The gathered information can then be utilized to manage urban infrastructure, including water supply, food services, environmental monitoring, and traffic congestion [29,30,31]. Smart agriculture is based on the IoT with future generation networks and is expected to benefit from the intelligently developed methodologies. The objectives of this strategy are to preserve water resources, lessen soil erosion, and improve soil quality [32,33]. An effective and scalable protocol for the remote monitoring and decision making of farms in rural areas is named the CL-IoT protocol, and it was proposed to focus on the requirements for smart farming applications [34]. To decrease network communication delay, latency, and energy consumption, cross-layer-based clustering, and routing algorithms were developed. The cluster head (CH) selection method based on cross-layers has been proposed as a means of solving the energy efficiency issue for resource constraint networks. Each sensor’s physical, medium access control (MAC), and network layer parameters were analyzed and chosen as the optimal CH for effective data transfer. The algorithm with a novel probabilistic decision rule that was inspired by nature is proposed and serves as a fitness function to choose the best path for data transfer. To choose the cluster head, a hybrid artificial neural network and decision tree method were built with the cognitive radio [35]. The base station receives more packets and collects more data from the typical sensor nodes as the residual energy level rises. The on-demand routing protocol is designed to hold data in local storage for retransmission during link failure to achieve reliable data transfer. Performance metrics for the proposed technique include throughput, packet drop rate, packet delivery ratio, normalized overhead, and residual energy. The effectiveness of the proposed strategy was compared to a cluster-based data aggregation scheme. A novel intelligent routing protocol was proposed in [36] to increase the network lifetime and offer energy efficiency in the routing process, which is used to deliver data to the irrigation system. The protocol is known as terrain-based routing using fuzzy rules for precision agriculture and it uses fuzzy rules to provide a revolutionary intelligent energy-efficient routing scheme. The routing decisions were made using the fuzzy inference method described in this work. The equalized cluster head election routing protocol and region-based routing are two routing algorithms that were constructed and compared with the system. The experimental findings demonstrate that the suggested algorithm outperforms the other available algorithms. For an Agriculture Internet of Things (AG-IoT) network, the authors proposed a supervised machine learning multipath and administrative-distance-based load balancing algorithm [37]. The proposed algorithm, also known as AI-enabled multi-hop and administrative-distance-based opportunistic routing (MHADBOR), processes the collected data from source to destination using the network’s multi-hop count and administrative-distance-based communication infrastructure. In addition, the authors frequently used CHs, microbase, and macrobase stations in the network to efficiently manage the deployed network traffic in a communication environment without congestion. In [38], the authors proposed an optimization of resource utilization in smart agriculture systems using IoT (SMAIoT) that can monitor several low-cost IoT sensor types. This framework gathers information from the soil, air, water, and insects and uses them to produce suitable decisions. The innovative aspect of the proposed framework is the scientific automation of functions such as irrigation, fertigation, pest detection, and pesticide spraying with efficient productivity. In [39], the authors provide an inter planetary file system (IPFS) storage for protecting agricultural sampling data based on the double-blockchain method for IoT networks. It stores the content of the sampled data using the IPFS network, and the proposed system can obtain the entire data segment using an oracle technique. Then, the authors developed a consortium blockchain, Agricultural Sample Data Chain (ASDC) by using Ethereum technology, and enhanced the Merkle Patricia Trie (MPT)-based accounts for all categories of sampled data. To retain a public record in the case of malicious attacks, block hashes are generated and uploaded on Ethereum’s main chain after storing the data in ASDC blocks.

### Limitations of the Existing Schemes

The summary of relevant studies shows that the IoT has grown significantly in creating and developing smart agriculture systems. It aids the farmers in monitoring soil conditions and water supply and increases productivity with the management of costs. It gathers the agricultural data and sends them to cloud databases to take the necessary actions accordingly. Numerous solutions have recently been proposed to deal with intelligent data monitoring with improved latency using machine learning techniques. However, many of the existing systems do not make it apparent how different connections are handled when there are redundant data. Moreover, efficient load balancing with the collaboration of mobile sensors is another significant research challenge [40,41]. Although many solutions have offered security for remote monitoring, this has been at the cost of computation and complexity. As a result, we need a framework to handle the timely supply of monitoring data using some smart and intelligent behaviors.

## 3. Material and Methods

This section presents a detailed discussion of the proposed framework with the system model. Additionally, its algorithm and developed components are explained.

### 3.1. System Model and Background of the Proposed Framework

**Figure 1:**
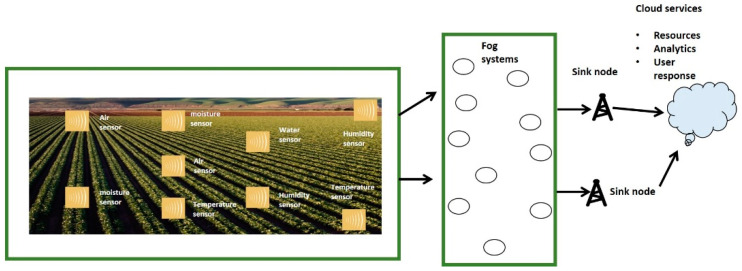
Scenario for fog-based IoT agricultural system.

The system model consists of sensors, fog nodes, and the sink node. They are deployed at random positions and the sink node is mobile. The fog nodes are considered more intelligent and have sufficient resources as compared to ordinary sensors. The mobile sink has a direct association with cloud systems. Initially, IoT sensors are arranged in an undirected graph G with finite vertices N and edges E. Each node has enough memory to hold and maintain its neighbors’ information. Similarly, neighbors in the fog system’s proximity are arranged in the form list based on a particular score. Fog systems not only aggregate the agricultural data for transmission towards the sink node but also offer a security layer among sensors and cloud services. Each node has a prefixed transmission range, and in case the sink node is far away from it, the proposed framework adopts a multi-hop forwarding decision. We show the scenario for the proposed framework in Figure 1. A robust routing strategy was built with the identification of the redundant nodes, and agricultural sensors are installed in the field to sense the various situations. In the case of redundant nodes, such information is not permitted for transmission. Later, fog layers are made up of numerous fog nodes to initiate communication with sink nodes. Agriculture users may simply obtain the data from their smart devices with the support of cloud platforms. Our proposed framework also ensures data privacy and security in an unpredictable environment.

**Figure 2:**
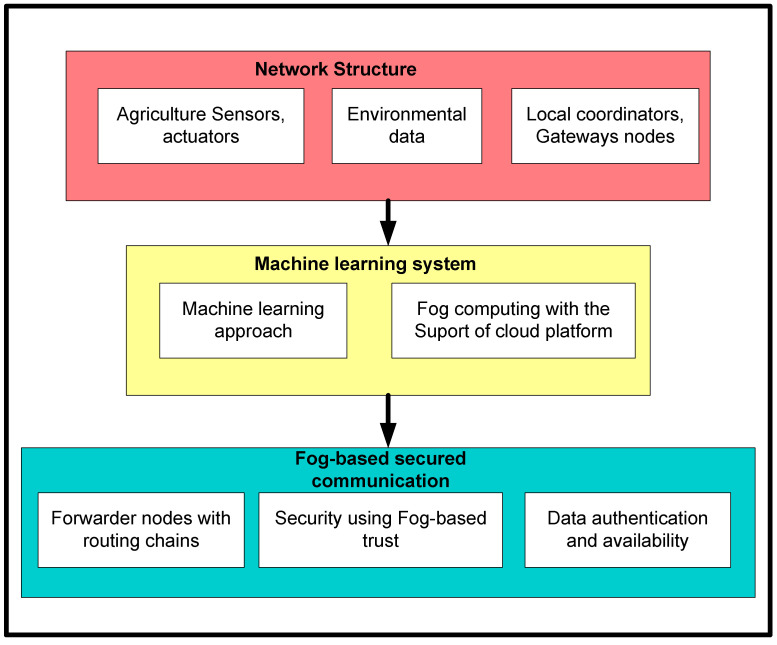
Block diagram of the proposed methodology.

Figure 2 depicts the block diagram of the proposed framework. It is comprised of three sub-blocks, i.e., network structure, machine learning system, and fog-based secured communication. Firstly, sensors, actuators, gateways, and other communication devices initialized themselves for sensing and forwarding agricultural data. The sensors continuously record environmental information, which is then sent to sink nodes via local coordinators or gateways. Second, a machine learning-based technique was created to reduce the communication overheads of the sensor nodes. Finally, the proposed system adopts the distributed regression function to assess the various attributes of the system and achieves efficient node management [42]. Moreover, the machine learning approach not only balances the load over the multiple routes, but the proposed framework can also identify the redundant nodes. Duplicate data are therefore prohibited from entering in proximity to fog nodes. In the end, a fog-based secured scheme is developed with the intelligence of fog nodes and cryptographic techniques. Fog nodes perform dual functionalities for communication with both the IoT system and cloud platform. This validates the authenticity of incoming packets, and accordingly, appropriate actions are taken. In the case of authorized nodes, they are allowed to send the data towards the cloud system or the request packets are dropped by the fog nodes. The information is recorded in the table about false messages and non-authentic devices.

### 3.2. Machine Learning-Based Distributed Regressional Analysis

Based on the undirected graph, the proposed framework identifies the initial and temporary routes $R(r_{1},\;r_{2},\ldots \ldots ,\;r_{n}$). The selected routes are based on the distance factor and each node maintains its neighbor table in a controlled manner. By exploring the neighbor table, each node formulates a route to the sink node for data transmission. To attain an efficient routing scheme with balances the load and bandwidth utilization, the proposed framework initiates the nodes management activity on the routes $r_{i}$. Let us consider that $N_{i}$ has a data $D_{i}$ to transmit to the sink node. Then, the proposed framework first identified the overlapping nodes that lay in the same transmission range. If any such nodes exist, then set their flag’s value in the routing table. The flag value indicates the forwarding status of the nodes. Let us suppose that $D_{t}$ is the distance threshold of the node NDs, and r is the predefined radius. To determine the overlapping nodes, the proposed framework exploits the search zone candidate nodes$\;C_{N}\;$based on the $D_{t}$ and r, as given below

(1) $$NDs\;\left(C_{N},\;D_{t}\right)\leq r$$

After determining the candidate nodes, the proposed framework updates the routing table of source node i with the latest statistical values of neighbors. Table 1 describes the format of the routing table. It comprised node identity, transmission power, flag value, computing score, and distance. The node identity which is one byte long is unique. The next field is one byte long and contains the value of preset transmission power. The flag field is just a Boolean parameter to indicate whether the node can be silent or not, and it is one bit long. Finally, the scoring factor is two bytes long and depends on the captured information about the nodes. In the end, the distance is 1 byte long and contains information about the space toward the fog system.

The proposed framework only allows one node in the transmission radius for sensing and forwarding the agricultural data. To achieve this, it determines the node score$\;sc_{i\;}$ with $w_{i\;}$ weighted coefficient using distributed weighted regression function X(i), and based on the maximum score, the flag value of the nodes i is set to either “*True*” or “*False*”, as given below:

(2) $$\{\begin{array}{c} if\;sc_{i}==max\\ f_{i\;}\;is\;\;True\\ false,\;otherwise \end{array}\;$$

(3) $$X\left(i\right)=w_{i\;}.\;sc_{i\;}$$

The $sc_{i\;}$ value for node i is determined by exploring the residual energy $e_{i\;}$, packets load $\;PL_{i\;}$, distance $\;Df_{i\;}$, as given below:

(4) sci=ei+1/PLi+1Dfi

where$\;PL_{i\;}\;$defines in terms of transmitted packets N at time interval T, as defined below.

(5) PLi=NT

### 3.3. Fog Systems-Based Security Maintenance

In the proposed framework, the fog nodes performed the role of the intermediate system between the data originating network and cloud services. Firstly, it received all the data and stores it in memory for further analysis and processing. It then confirms the data authenticity to forward it towards the cloud system in the multi-hop discipline. Then, the aggregated data$\;D_{A\;}$ is encrypted En using the secret key k of the node i. Additionally, it is integrated with the identity $ID_{i}\;$as given below.

(6) $$En\left(k\right)\;(D_{A}+ID_{i})$$

Upon receiving, fog nodes decrypt it to retrieve the aggregated data and the identity of the data-originated node. After the decryption process, the fog nodes verify the identity of the nodes with their stored information, and if it matches positively, then it will look up its routing table for the selection of the next hop among the neighboring fogs. Moreover, the cloud system c authenticates the receiving data using digital signatures. To achieve this, the fog node FG first digitally signs the aggregated data $D_{A\;}$ using its private key $p_{r\;}$ to generate a secret value S, as given below.

(7) $$FG_{c\;}=S\;+D_{A\;}$$

where

(8) $$E_{p_{r}}\left(D_{A}\right)=S$$

**Figure 3:**
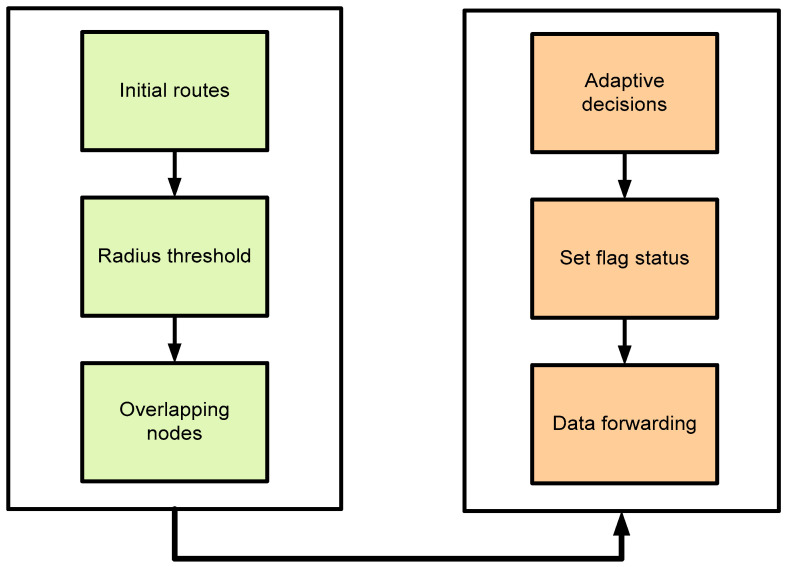
Developed methods for the proposed framework.

On the other side, the cloud system first authenticates the digital signature using the public key of the fog nodes and upon successful verification, it is further forwarded to the connected end users with the IoT system. The flow between the developed techniques for intelligent agricultural routing is shown in Figure 3. Initial routes are formulated using the greedy method and exploited for optimizing the routing process. It can detect redundant nodes in the proximity of a predefined radius and accordingly, it sets the flag value by exploring the intelligent technique of machine learning. The flag value indicates the status of neighbors and whether they can transmit the data or keep them in silent mode. Finally, agricultural data is transmitted towards the sink node using the fog-based IoT system.

**Figure 4:**
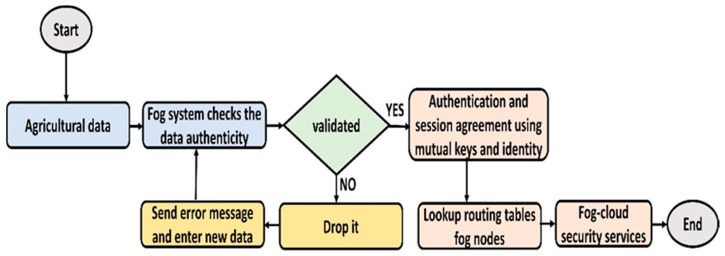
Flowchart of the proposed security algorithm.

Figure 4 explains the security algorithm for transmitting agricultural data with the support of fog nodes. It clarifies the fog-based trust mechanism with privacy concerns and reliability. Accordingly, the proposed security algorithm decreases the probabilities of network attacks and stabilizes the communication system. Fog nodes are utilized as a bridge to facilitate both the IoT and cloud systems. Firstly, fog nodes verified the incoming agricultural data, and upon its validation, authentication and session agreement are established. The session agreement is valid for a particular time and needs to be refreshed later for further communication. In case verification is unsuccessful, then the error message is generated towards the data originating node. Additionally, fog nodes construct routing paths for data forwarding by exploring the routing table. The routing information is updated and evaluated each time that data transmission is needed. In the end, fog and cloud ensure data security using cryptography-based digital signature and encryption techniques. Such a technique provides high-level security measures to the upper layers.

**Figure 5:**
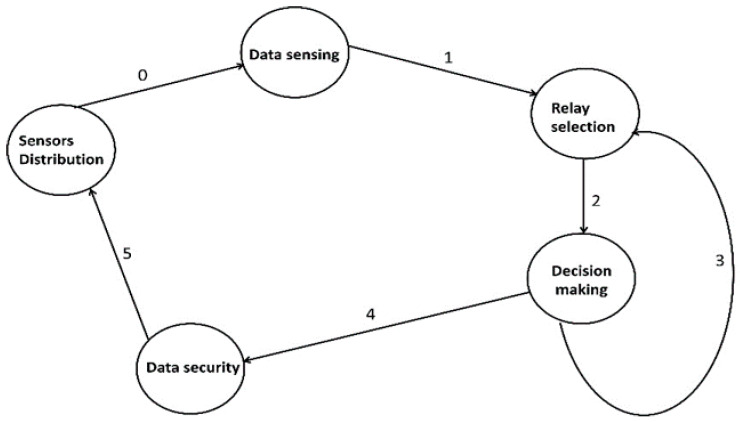
States of the proposed framework.

Moreover, the functioning of the proposed framework consists of many states, as depicted in Figure 5. The system will be in a specific state at a given time, and it will change states when a particular trigger is called. The objectives of the states are defined below.

- *Sensor’s deployment:* In this state, agriculture nodes are randomly dispersed in the targeted area. They have limited constraints and not enough memory, transmissions, and processing resources. They are not able to communicate with the sink node directly;
- *Data sense:* All the deployed sensors, actuators, and IoT devices collaborate in collecting the data. In addition, the data are transferred to the sink nodes through integrating fog systems. The fog nodes are explored for reducing communication delays;
- *Relay node:* The data are obtained from the deployed sensors and forwarded towards the relay node. The roles for relay nodes are not predefined, they are chosen using quality-aware parameters;
- *Decision making:* In this state, a distributed machine learning technique is applied using network statistics to offer the optimal results for attaining a reliable communication system;
- *Data security:* In this end, security actions are performed in this state. It identifies faulty nodes and false messages to detect the comprised data using private values. It increases the reliability of remote users that are connected to cloud systems.

## 4. Simulations

This section presents the simulation environment and a discussion of the various tests. We ran the simulations of a core i7 laptop with 64 GB RAM and 1 TB hard drive. 20 trials of the simulations were performed. Between 150 and 750 sensor nodes were used in each simulation experiment. Zigbee technologies were used in the testing, and experiments were carried out in 1000 m × 1000 m area with a variety of configuration settings. For sensing the agricultural environment, numerous sensor categories were utilized, including temperature, air humidity, soil moisture, and water quality. Two sink nodes, between ten and fifteen 15 fog nodes, and various relay nodes were also deployed. Sensor nodes collected the agriculture data and forwarded them to the relay nodes for aggregation and data routing. The fog nodes existed between sensors and sink nodes. The duration of the experiments was 2000 rounds and each round had a time interval of 20 s. The sensors were equipped with GPS. Communication channels were scattered with certain malicious nodes to test the system’s security. The results analysis was obtained based on the packet delivery, energy efficiency, network connectivity, and reliability metrics. All the network metrics were evaluated under scenarios of varying sensors and distances from the sink node. Table 2 contains the various parameters for simulations.

## 5. Results with Discussion

**Figure 6:**
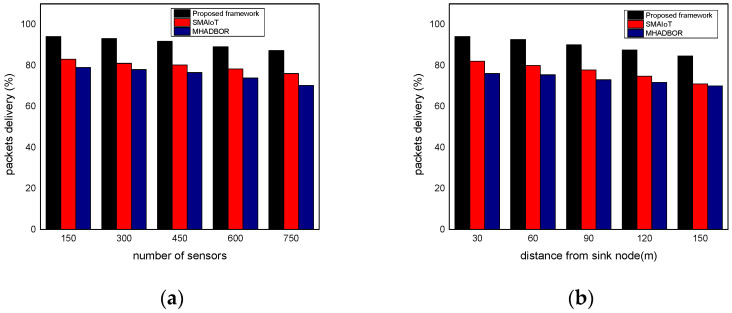
(**a**) Packets delivery with varying sensors and (**b**) Packets delivery with varying distance.

**Figure 7:**
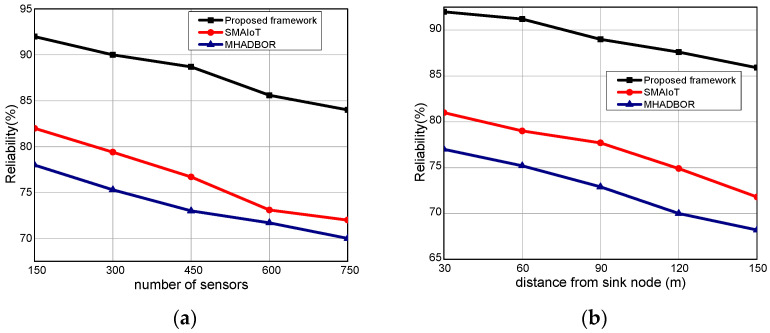
(**a**) Reliability with varying sensors and (**b**) Reliability with varying distance.

**Figure 8:**
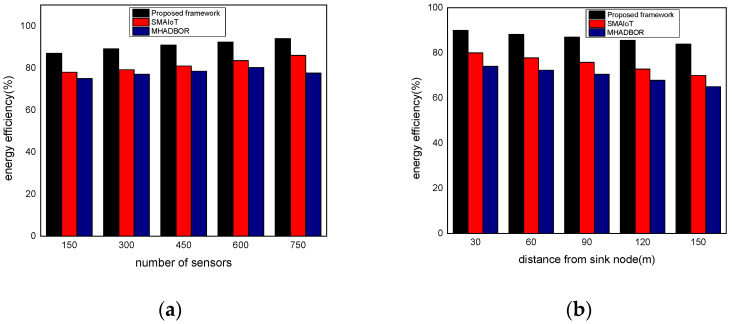
(**a**) Energy efficiency with varying sensors and (**b**) Energy efficiency with varying distance.

**Figure 9:**
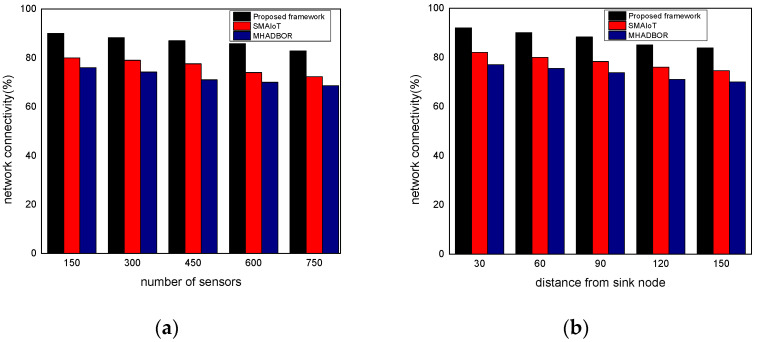
(**a**) Network connectivity with varying sensors and (**b**) Network connectivity with varying distance.

We evaluated the performance of the proposed framework with other studies in terms of energy efficiency. The performance evaluation of the proposed framework against those of related works is shown in Figure 6a,b for varied IoT sensors and the distance from the sink node. It was found that the proposed framework, even in the presence of malfunctioning nodes, considerably improves the delivery rate of data packets by an average of 20% for varying nodes, and 22% for varying distances from the sink. It results from exploring security approaches, key generation, and mutual trust. In the proposed paradigm, the intelligent system achieves sustainability and effectively identifies harmful activities by utilizing the secret and personal data of devices. Furthermore, the sink nodes are more powerful than IoT sensors and verify each activity before sending it to the end users. Unlike the majority of the proposed research, our framework explicitly provides intelligent decision modules for enhancing packet delivery performance with the aid of machine learning and sustains the network load with improved throughput. Figure 7a,b demonstrate the comparison of the proposed framework in the literature and it was discovered that it notably improves the network reliability by an average of 18% and 23% for both scenarios. This is due to the exposure of machine learning techniques to identify the optimal IoT nodes and intelligently interact with cloud systems. Moreover, the proposed framework can tackle redundant information and decreases the chances of data unavailability and congestion. Additionally, the security solution decreases unwanted traffic across the open transmission system and stops malicious devices from sending false route request packets. As a result, the proposed framework lengthens the response time for critical situations with a nominal delay rate. The comparison of the proposed and existing solutions is revealed in Figure 8a,b in terms of varying nodes and distances from the sink. The statistical analysis demonstrated that the proposed framework increased energy usage by 15% and 24%, respectively. Energy efficiency was found to be negatively impacted when the number of devices increased. On the other hand, the proposed framework provides a smart energy solution based on a distributed machine learning technique and smoothly selects the updated routes by utilizing the optimum solution. Additionally, the proposed framework effectively defends against multiple attacks and minimizes the consumption of network bandwidth by employing a security algorithm. Consequently, the nodes’ energy is increased, which improves the performance of the entire network. By finding the redundant nodes in the routing table, the flag status is modified. Nodes whose flag value is false are therefore prohibited from participating in data routing. The performance comparison of the proposed framework to the existing solutions for network connectivity is illustrated in Figure 9a,b. Network connectivity indicates the active time at which nodes use particular communication links. The connectivity ratio for the proposed framework against various IoT devices and varied distances is remarkably enhanced by an average of 16% for varying distances from the sink and 20% in terms of varying nodes. This is due to the ability of the proposed framework to efficiently manage power distribution across the sensors and investigate the machine learning principles for accomplishing forwarding decisions. Moreover, the proposed framework directs the routing module to formulate the routes by re-evaluating the decisions whenever any disrupted intermediate links are discovered or frequent re-transmissions. The proposed framework successfully manages the transmission overheads by including the least computational cost function.

## 6. Conclusions

With an emphasis on green energy and remote monitoring, smart technologies and agricultural systems have grown significantly in recent years. It controls the plants, soil characteristics, water pressure, and weather-related information, which benefits farmers and increases production. However, limited solutions have been proposed to enhance production in precision agriculture while reducing latency and information disturbance. Furthermore, protecting agricultural data while utilizing the insecure Internet is another significant research challenge. In this study, we presented a framework for providing a farmer communication system to enhance timely delivery through the cooperation of fog systems. This system also incorporates redundant information detection, which reduces network bandwidth inefficiencies. Additionally, the proposed framework employs security methods to counter privacy attacks on sensing data. The performance results showed the good outcomes of the proposed framework with maximum energy efficiency and delivery ratio. Furthermore, its performance has proven the improved processing usage in the existence of malicious devices. In the future, we intend to cope with the proposed framework’s scalability and load-balancing issues with the support of a multi-cloud architecture. Moreover, we would like to integrate security to maintain cloud integrity from the point of users’ perspective.

## Figures and Tables

**Table 1:** Format of a routing table.

1 Byte	1 Byte	1 Bit	2 Bytes	1 Byte
Node identity, id	$\mathrm{Transmission}\;\mathrm{power}\;t_{x\;}$	$\mathrm{Flag}\;\mathrm{value},\;f_{i\;}$	$\mathrm{Score},\;sc_{i\;}$	$\mathrm{Distance}\;\mathrm{to}\;\mathrm{fog}\;\mathrm{nodes},\;\;Df_{i\;}$

**Table 2:** Simulation parameters.

Parameter	Value
Agriculture sensors	Varying 150–750
Sensor’s type	Temperature, air humidity, water quality, soil moisture
Fog nodes	10–15
Interface	IEEE 802.15.4
Round step	20 s
Sink nodes	2
Number of rounds	2000
Initial energy	5 J
Field dimension	1000 × 1000
Simulations	20

## Data Availability

All data are provided within the manuscript.

## References

[1] Zhou I., Makhdoom I., Shariati N., Raza M.A., Keshavarz R., Lipman J., Abolhasan M., Jamalipour A. (2021). Internet of things 2.0: Concepts, applications, and future directions. IEEE Access.

[2] Nguyen V.-L., Lin P.-C., Cheng B.-C., Hwang R.-H., Lin Y.-D. (2021). Security and Privacy for 6G: A Survey on Prospective Technologies and Challenges. IEEE Commun. Surv. Tutor..

[3] Rehman A., Haseeb K., Saba T., Lloret J., Ahmed Z. (2021). Mobility Support 5G Architecture with Real-Time Routing for Sustainable Smart Cities. Sustainability.

[4] Rehman A., Haseeb K., Saba T., Lloret J., Tariq U. (2021). Secured Big Data Analytics for Decision-Oriented Medical System Using Internet of Things. Electronics.

[5] Quy V.K., Hau N.V., Anh D.V., Quy N.M., Ban N.T., Lanza S., Randazzo G., Muzirafuti A. (2022). IoT-Enabled Smart Agriculture: Architecture, Applications, and Challenges. Appl. Sci..

[6] Lloret J., Garcia M., Bri D., Sendra S. (2009). A Wireless Sensor Network Deployment for Rural and Forest Fire Detection and Verification. Sensors.

[7] Gavrilović N., Mishra A. (2021). Software architecture of the internet of things (IoT) for smart city, healthcare and agriculture: Analysis and improvement directions. J. Ambient Intell. Humaniz. Comput..

[8] Raj M., Gupta S., Chamola V., Elhence A., Garg T., Atiquzzaman M., Niyato D. (2021). A survey on the role of Internet of Things for adopting and promoting Agriculture 4.0. J. Netw. Comput. Appl..

[9] Haseeb K., Saba T., Rehman A., Ahmed Z., Song H.H., Wang H.H. (2022). Trust management with fault-tolerant supervised routing for smart cities using internet of things. IEEE Internet Things J..

[10] Khan A.I., Alsolami F., Alqurashi F., Abushark Y.B., Sarker I.H. (2022). Novel energy management scheme in IoT enabled smart irrigation system using optimized intelligence methods. Eng. Appl. Artif. Intell..

[11] Singh R.K., Berkvens R., Weyn M. (2021). AgriFusion: An Architecture for IoT and Emerging Technologies Based on a Precision Agriculture Survey. IEEE Access.

[12] Maddikunta P.K.R., Hakak S., Alazab M., Bhattacharya S., Gadekallu T.R., Khan W.Z., Pham Q.-V. (2021). Unmanned Aerial Vehicles in Smart Agriculture: Applications, Requirements, and Challenges. IEEE Sens. J..

[13] Farooq M.S., Riaz S., Abid A., Abid K., Naeem M.A. (2019). A Survey on the Role of IoT in Agriculture for the Implementation of Smart Farming. IEEE Access.

[14] Johnson N., Kumar M.S., Dhannia T. A study on the significance of smart IoT sensors and Data science in Digital agriculture. Proceedings of the 2020 Advanced Computing and Communication Technologies for High Performance Applications (ACCTHPA).

[15] He S., Shi K., Liu C., Guo B., Chen J., Shi Z. (2022). Collaborative Sensing in Internet of Things: A Comprehensive Survey. IEEE Commun. Surv. Tutor..

[16] Churcher A., Ullah R., Ahmad J., Rehman S.U., Masood F., Gogate M., Alqahtani F., Nour B., Buchanan W. (2021). An Experimental Analysis of Attack Classification Using Machine Learning in IoT Networks. Sensors.

[17] Bagaa M., Taleb T., Bernabe J.B., Skarmeta A. (2020). A Machine Learning Security Framework for Iot Systems. IEEE Access.

[18] Demestichas K., Peppes N., Alexakis T. (2020). Survey on Security Threats in Agricultural IoT and Smart Farming. Sensors.

[19] Elijah O., Rahman T.A., Orikumhi I., Leow C.Y., Hindia M.N. (2018). An Overview of Internet of Things (IoT) and Data Analytics in Agriculture: Benefits and Challenges. IEEE Internet Things J..

[20] Abunadi I., Mengash H.A., Alotaibi S.S., Asiri M.M., Hamza M.A., Zamani A.S., Motwakel A., Yaseen I. (2022). Optimal Multikey Homomorphic Encryption with Steganography Approach for Multimedia Security in Internet of Everything Environment. Appl. Sci..

[21] Ferrag M.A., Shu L., Yang X., Derhab A., Maglaras L. (2020). Security and privacy for green IoT-based agriculture: Review, blockchain solutions, and challenges. IEEE Access.

[22] Vangala A., Das A.K., Chamola V., Korotaev V., Rodrigues J.J. (2022). Security in IoT-enabled smart agriculture: Architecture, security solutions and challenges. Clust. Comput..

[23] Haseeb K., Ud Din I., Almogren A., Islam N. (2020). An Energy Efficient and Secure IoT-Based WSN Framework: An Application to Smart Agriculture. Sensors.

[24] Rao P.M., Deebak B.D. (2022). Security and privacy issues in smart cities/industries: Technologies, applications, and challenges. J. Ambient Intell. Humaniz. Comput..

[25] Singh T., Solanki A., Sharma S.K., Nayyar A., Paul A. (2022). A Decade Review on Smart Cities: Paradigms, Challenges and Opportunities. IEEE Access.

[26] Sodhro A.H., Pirbhulal S., Luo Z., de Albuquerque V.H.C. (2019). Towards an optimal resource management for IoT based Green and sustainable smart cities. J. Clean. Prod..

[27] Abidoye A.P., Kabaso B. (2021). Energy-efficient hierarchical routing in wireless sensor networks based on fog computing. EURASIP J. Wirel. Commun. Netw..

[28] Wang T., Zhang G., Alam Bhuiyan Z., Liu A., Jia W., Xie M. (2020). A novel trust mechanism based on Fog Computing in Sensor–Cloud System. Future Gener. Comput. Syst..

[29] Ahmed S., Hossain F., Kaiser M.S., Noor M.B.T., Mahmud M., Chakraborty C. (2021). Artificial Intelligence and Machine Learning for Ensuring Security in Smart Cities. Data-Driven Mining, Learning and Analytics for Secured Smart Cities.

[30] Alam T. (2021). Cloud-Based IoT Applications and Their Roles in Smart Cities. Smart Cities.

[31] Aldegheishem A., Alrajeh N., Garcia L., Lloret J. (2022). SWAP: Smart WAter Protocol for the Irrigation of Urban Gardens in Smart Cities. IEEE Access.

[32] Zikria Y., Ali R., Afzal M., Kim S. (2021). Next-Generation Internet of Things (IoT): Opportunities, Challenges, and Solutions. Sensors.

[33] Maksimovic M. (2018). Greening the future: Green Internet of Things (G-IoT) as a key technological enabler of sustainable develop-ment. Internet of Things and Big Data Analytics Toward Next-Generation Intelligence.

[34] Mahajan B.H., Badarla A., Junnarkar A.A. (2021). CL-IoT: Cross-layer Internet of Things protocol for intelligent manufacturing of smart farming. J. Ambient. Intell. Humaniz. Comput..

[35] Tabassum M., Perumal S., Kashem S.B.A., Ponnan S., Chakraborty C., Chowdhury M.E.H., Khandakar A. (2022). Enhance data availability and network consistency using artificial neural network for IoT. Multimedia Tools Appl..

[36] Pandiyaraju V., Logambigai R., Ganapathy S., Kannan A. (2020). An Energy Efficient Routing Algorithm for WSNs Using Intelligent Fuzzy Rules in Precision Agriculture. Wirel. Pers. Commun..

[37] Adil M., Khan M.K., Jamjoom M., Farouk A. (2021). MHADBOR: AI-Enabled Administrative-Distance-Based Opportunistic Load Balancing Scheme for an Agriculture Internet of Things Network. IEEE Micro.

[38] Jani K.A., Chaubey N.K. (2021). A Novel Model for Optimization of Resource Utilization in Smart Agriculture System Using IoT (SMAIoT). IEEE Internet Things J..

[39] Ren W., Wan X., Gan P. (2020). A double-blockchain solution for agricultural sampled data security in Internet of Things network. Futur. Gener. Comput. Syst..

[40] Dilek S., Irgan K., Guzel M., Ozdemir S., Baydere S., Charnsripinyo C. (2022). QoS-aware IoT networks and protocols: A comprehensive survey. Int. J. Commun. Syst..

[41] Khan S., Parkinson S., Qin Y. (2017). Fog computing security: A review of current applications and security solutions. J. Cloud Comput..

[42] Kumar D.P., Amgoth T., Annavarapu C.S.R. (2019). Machine learning algorithms for wireless sensor networks: A survey. Inf. Fusion.

